# 

**Published:** 1854-06

**Authors:** 


Vol. I. JUNE, 1854. No. 11.

### IOWA MEDICAL JOURNAL

CONDUCTED BY THE FACULTY OF THE

**Medical Department of the Iowa University.**

PUBLISHED MONTHLY.

TERMS--TWO DOLLARS, IN ADVANCE.

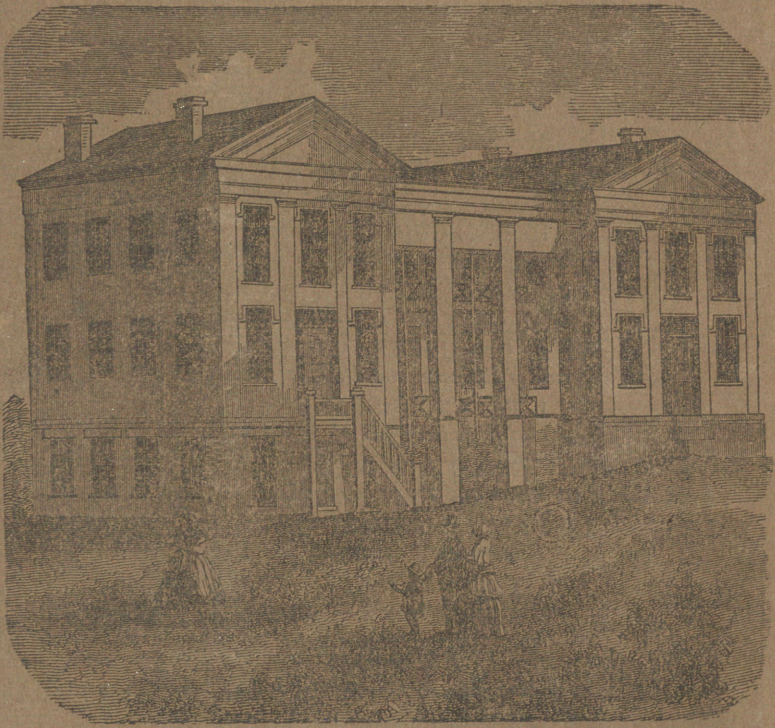

KEOKUK, IOWA:

Printed at the Whig Book and Job Office.

ADDRESS ALL COMMUNICATIONS, POST-PAID, TO "MEDICAL COLLEGE, BOX 109, KEOKUK, IOWA."

